# Effects of Notch signalling on the expression of SEMA3C, HMGA2, CXCL14, CXCR7, and CCL20 in breast cancer

**DOI:** 10.3906/biy-1808-58

**Published:** 2019-02-07

**Authors:** Cansu KÜÇÜKKÖSE, Özden YALÇIN ÖZUYSAL

**Affiliations:** 1 Department of Molecular Biology and Genetics, Faculty of Science, İzmir Institute of Technology , İzmir , Turkey

**Keywords:** Breast cancer, metastasis, Notch signalling, SEMA3C, HMGA2, CXCL14, CXCR7, CCL20

## Abstract

Metastasis is the main reason for death in breast cancer. Understanding the molecular players in metastasis is crucial for diagnostic and therapeutic purposes. Notch signalling plays an oncogenic role in breast tumorigenesis and is involved in metastasis. Downstream mediators of Notch signalling in prometastatic processes are not yet fully discovered. Here we aimed to investigate whether Notch signalling regulates the expression of SEMA3C, HMGA2, CXCL14, CXCR7, and CCL20, which are involved in prometastatic processes, in breast cell lines. To this end, expression of the selected genes was analysed following Notch activation by overexpression of the Notch1 intracellular domain in the normal breast epithelial cell line MCF10A, and inhibition by silencing of the Notch transcriptional mediator RBPjκ in the breast cancer cell line MDA MB 231. SEMA3C and HMGA2 mRNA were decreased, while CXCL14 and CXCR7 mRNA were increased significantly in response to Notch activation in MCF10A cells. Notch inhibition in MDA MB 231 cells significantly decreased HMGA2 and CCL20 mRNA. Protein levels were not significantly altered by Notch modulation. In conclusion, we showed that Notch signalling regulates expression of SEMA3C, CXCL14, CCL20, CXCR7, and HMGA2, which are prominent candidate genes that might function downstream of Notch to induce prometastatic processes.

## 1. Introduction

Breast cancer is the second most frequently diagnosed cancer, comprising 25% of all cancer diagnoses worldwide. Despite improvements in early detection and treatment approaches, breast cancer is still the leading cause of cancer-related deaths in women (Ferlay et al., 2013) . While 62% of breast cancer cases are localised, 31% have regional and 6% have distant metastasis at the time of diagnosis. Five-year survival rates for patients with localised tumours or tumours with regional metastasis are 98.9% and 85.2%, respectively. However, the survival rate dramatically falls to 26.9% for patients with distant metastasis (Howlader et al., 2017) . The main reason for breast-cancer-related deaths is metastasis, for which there are no effective treatment approaches. Thus, understanding the key molecular players in breast cancer metastasis is crucial for diagnostic and therapeutic purposes.

Notch is an oncogenic signalling pathway involved in breast cancer. Notch receptors (Notch 1–4 in mammals) are transmembrane proteins that go through two subsequent cleavages by gamma-secretase following the binding of transmembrane ligands (Delta-like ligand (Dll) 1, 3, 4 and Jagged 1, 2) inserted into the membrane of the neighbouring cells. The cleavages release the Notch intracellular domain (NICD), which translocates to the nucleus and activates its target genes by binding to its specific mediator, RBPjk, a transcription factor. Notch 4 was first discovered as one of the integration sites of mouse mammary tumour virus (MMTV), which results in continuous expression of the Notch4 intracellular domain and mammary tumour formation (Gallahan and Callahan, 1997). Since then, Notch activation has been shown to induce cell proliferation and transformation of breast cells, cause mammary tumour formation in transgenic mouse models, and correlate with poor prognosis in breast cancer (Guo et al., 2011).

Notch signalling is involved in the regulation of epithelial to mesenchymal transition (EMT), migration, and invasion, which are considered as initial steps of metastasis (Guo et al., 2011; Espinoza and Miele, 2013) . In different cancer types, including glioma, hepatocellular carcinoma, and lung and pancreas tumours, Notch activation induces EMT through transcription factors Snail-1, Snail-2, and Twist, which are EMT regulators (Bao et al., 2011; Matsuno et al., 2012; Wang et al., 2012; Zhang et al., 2012) . In breast cancer, several factors such as radiation, hypoxia, and Klf4 induce EMT, migration, and invasion via activating Notch receptors (Chen et al., 2010; McGowan et al., 2011; Xing et al., 2011; Kim et al., 2016) . In contrast, gamma-secretase inhibitors and Numb, which are negative regulators of Notch signalling, suppress these processes through inhibition of Notch signalling (McGowan et al., 2011; Zhang et al., 2016) .

Although Notch signalling was shown to interact with several molecules including TGFβ, IL6/STAT3, and microRNAs mir4c and mir200c to exert its prometastatic function, its downstream mediators are not yet fully discovered (Studebaker et al., 2008; Zhang et al., 2010; Brabletz et al., 2011; Yang et al., 2011; Hsu et al., 2012; Yu et al., 2012) . In this respect, in order to determine novel Notch target genes in breast cells, we analysed the list of genes that were shown to be differentially expressed in microarray analysis in response to Notch activation in the normal breast cell line MCF10A (Mazzone et al., 2010) . Among the most significantly altered 1000 genes we selected 5, SEMA3C, HMGA2, CXCL14, CXCR7, and CCL20, which are known to be involved in prometastatic processes but whose interaction with Notch had not been investigated. Here we aimed to investigate whether Notch signalling regulates the expression of these genes in breast cell lines.

## 2. Materials and methods

### 2.1. Cell culture and gene expression

The normal breast epithelial cell line MCF10A and the breast cancer cell line MDA MB 231 were obtained from ATCC. MCF10A cells were cultured in DMEM/ F12 including HEPES (25 mM), epidermal growth factor (20 ng/mL), cholera toxin (100 ng/mL), hydrocortisone (500 ng/mL), 5% horse serum, and insulin (10 µg/mL). MDA MB 231 cells were cultured in DMEM with 10% foetal bovine serum. Cells were grown with 5% CO2 at 37 °C. cDNA of the Notch1 intracellular domain (NICD) was overexpressed by MSCV-NICD retrovirus in order to activate Notch signalling (Zengin et al., 2015) . As the negative control, empty MSCV virus was used. shRNA against RBPjk, the mediator of canonical Notch signalling, was expressed by lentivirus to inhibit Notch activity (Procopio et al., 2015; Zengin et al., 2015) . As the negative control, shRNA against green uflorescent protein (GFP) was expressed by lentivirus. Virus preparation and infection were done as described previously (Zengin et al., 2015) . Briefly, viruses were collected from supernatant of 293T cells transfected with viral backbone, packaging, and envelope plasmids. The supernatants that contain the virus were collected and their titres were checked. Only the virus preparations that had similar titres were used for the experiments. All the analyses were done 48 h after infection.

### 2.2. RNA isolation and QRT-PCR

Total RNA was isolated with a PureLink RNA Isolation Kit (Invitrogen), and cDNA synthesis was done using a RevertAid First Strand cDNA Synthesis Kit (Fermentas). SYBR Green Master Mix (Fermentas) was used for real-time RT-PCRs (QRT-PCR) done on an iCycler (Bio-Rad). Three independent experiments were performed and average values ± SD (standard deviation) were represented. TATA box-binding protein (TBP) was used as the endogenous control gene. Statistical significance was calculated by twotailed Student’s t-test. The primer pairs for each gene were as follows: CCL20 5’- GTCTGTGTGCGCAAATCCAA -3’, 5’- GACAAGTCCAGTGAGGCACA -3’; CXCR7 5’- TGTGGGTTACAAAGCTGCCA -3’, 5’- GAGGCGGGCAATCAAATGAC -3’; CXCL14 5’- AAGGGACCCAAGATCCGCTA -3’, 5’- GACACGCTCTTGGTGGTGAT -3’; HEY2 5’-AAGATGCTTCAGGCAACAGG-3’, 5’-GCACTCTCGGAATCCTATGC-3’; HMGA2 5’- GCCCTCTCCTAAGAGACCCA -3’, 5’CTGCCTCTTGGCCGTTTTTC -3’; SEMA3C 5’- ACCAAGAGGAATGCGGTCAG -3’, 5’TGTTGACAAGGCTACGCAGT -3’; TBP 5’- TAGAAGGCCTTGTGCTCACC -3’, 5’TCTGCTCTGACTTTAGCACCTG -3’.

### 2.3. Protein isolation and western blot

RIPA buefr was used for protein isolation. First 20–100 µg of total protein was run on SDS/PAGE and then transferred to PVDF membranes. Rabbit anti-Hey2 (1:500, Abcam, AB184246), rabbit anti-CXCR7 (1:500, Abcam, AB38089), rat anti-SEMA3C (1:500, Abcam, AB135167), and rabbit anti-β-actin (1:1000, Abcam, AB75186) were used for immunoblotting. β-Actin was used for equal loading control. Quantification of the western blot images was done with the “Gels” tool of ImageJ. For each independent experiment, signal intensities of the analysed proteins were first normalised to β-actin levels for each condition and then NICD infected samples were normalised to control infected samples. Then the average values of three independent experiments were represented for each protein analysed. Statistical significance was calculated by two-tailed Student’s t-test.

## 3. Results

### 3.1. Effects of Notch activation on the mRNA and protein expression of candidate genes

**Figure 1:**
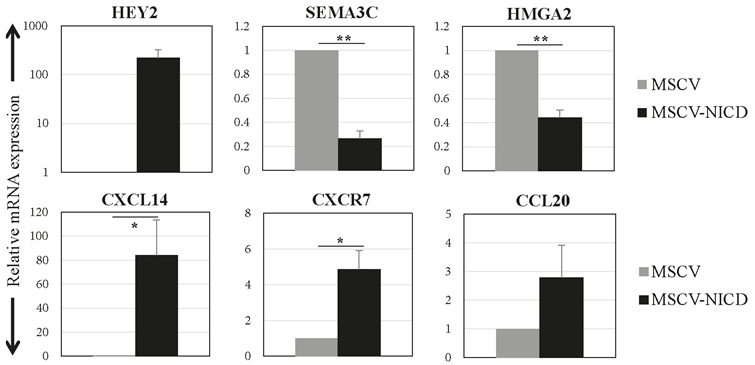
mRNA expression levels of the candidate genes in response to Notch activation. Relative mRNA expression levels of candidate genes and Notch target HEY2 were analysed 48 h after infection of MCF10A cells with control (MSCV) or active Notch1 receptor expressing virus (MSCV-NICD). Averages of three independent experiments are shown. Error bars represent standard deviation (P values: *: < 0.05, **: < 0.0005).

MCF10A is a normal breast cell line that does not have endogenous Notch signalling activity. Notch activation in MCF10A cells results in transformation demonstrated by increased colony formation in soft agar and resistance to apoptosis. Further, MCF10A cells with active Notch signalling acquired a more elongated mesenchymal-like phenotype and reduced E-cadherin expression, which suggests that Notch activation could induce a prometastatic phenotype in these cells (Stylianou et al., 2006) . Thus, we selected MCF10A cells to test the effects of Notch activation on the expression of selected genes. Notch signalling activation was achieved by overexpression of the Notch1 intracellular domain (NICD) via infection of MCF10A cells with the virus expressing corresponding cDNA (MSCVNICD). mRNA expression of Notch target gene HEY2 was increased by more than 200-fold in MSCV-NICD infected cells compared to control (MSCV) infected cells in 48 h, showing that Notch signalling activation was successful (Figure 1). SEMA3C and HMGA2 expression levels were significantly reduced by 73% and 45%, respectively. CXCL14 and CXCR7 mRNA levels were significantly increased by 64- and 5-fold, respectively. CCL20 mRNA expression was increased by 2.8-fold, which did not reach statistical significance (Figure 1).

**Figure 2:**
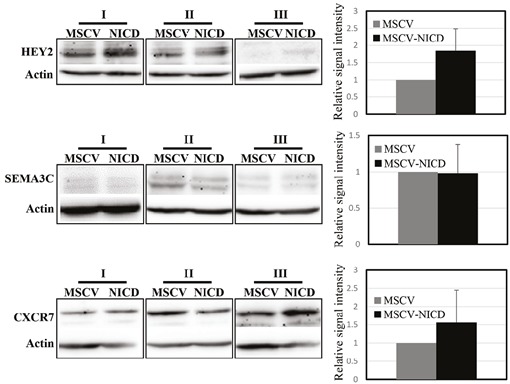
Protein expression of SEMA3C and CXCR7 in response to Notch activation. Protein expression levels of SEMA3C, CXCR7, and Notch target gene, HEY2, were analysed 48 h after infection of MCF10A cells with control (MSCV) or active Notch1 receptor expressing virus (MSCV-NICD). Actin was used as equal loading control. Averages of three independent experiments are shown. Error bars represent standard deviation.

Protein expression levels were analysed by western blot 48 h after infection of MCF10A cells. Protein levels of the Notch target gene HEY2 were upregulated around 2-fold, which confirmed activation of Notch signalling in MCF10A cells (Figure 2). Although CXCR7 protein levels had a tendency to increase upon Notch activation, we did not observe a significant change in the protein levels of either SEMA3C or CXCR7 (Figure 2).

### 3.2. Effects of Notch inhibition on the mRNA and protein expression of candidate genes

**Figure 3:**
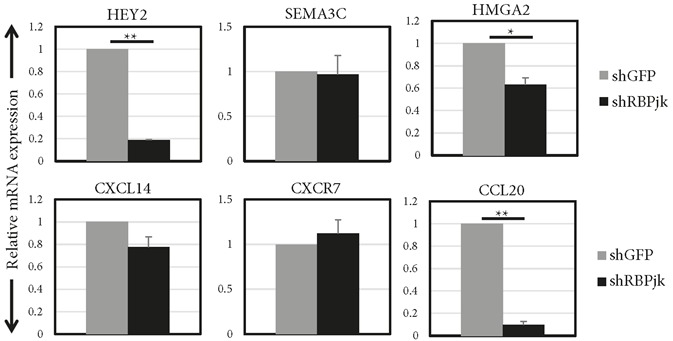
mRNA expression levels of candidate genes in response to Notch inhibition. Relative mRNA expression levels of candidate genes and Notch target HEY2 were analysed 48 h after infection of MDA MB 231 cells with control virus (shGFP) or virus expressing shRNA against RBPjk (shRBPjk). Averages of three independent experiments are shown. Error bars represent standard deviation. (P values: *: <0.0005, **: <0.000005).

Notch signalling was inhibited in MDA MB 231 cells, which have high endogenous Notch activity, by silencing RBPjk, the transcriptional mediator of Notch receptors. Silencing RBPjk (shRBPjk) downregulated the Notch target gene HEY2 significantly by 81% compared to the control group (shGFP) (Figure 3). HMGA2 and CCL20 mRNA expression levels were significantly decreased by 36% and 90%, respectively. SEMA3C and CXCR7 mRNA expression levels were not affected by Notch inhibition, while CXCL14 mRNA had a tendency to be reduced but the value did not reach statistically significant levels (Figure 3).

**Figure 4:**
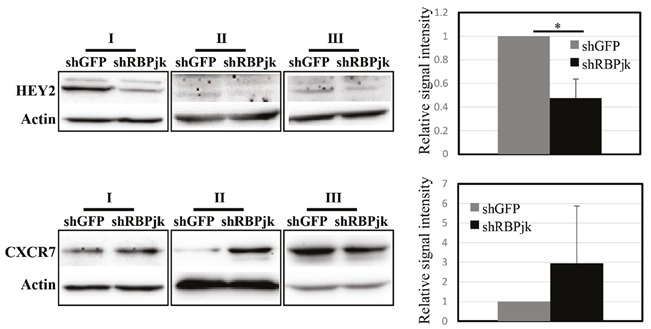
CXCR7 protein expression in response to Notch inhibition. CXCR7 and HEY2 proteins were analysed 48 h after infection of MDA MB 231 cells with control virus (shGFP) or virus expressing shRNA against RBPjk (shRBPjk). Actin was used as equal loading control. Averages of three independent experiments are shown. Error bars represent standard deviation (P value: *: <0.002).

HEY2 protein levels were reduced by 50% 48 h after the infection of MDA MB 231 cells with virus expressing shRNA against RBPjk (Figure 4). In two out of the three experiments, there was increased signal intensity for CXCR7 protein in response to Notch inhibition; however, the overall change was not statistically significant (Figure 4).

## 4. Discussion

In the present study, we investigated how Notch signalling activity effects the expression of SEMA3C, HMGA2, CXCL14, CXCR7, and CCL20 in breast cell lines, in order to define candidate genes that could be involved in prometastatic functions of Notch signalling.

SEMA3C, which is a secreted protein that belongs to class 3 of the semaphorin family, was found in two different forms, long and short. The long form of SEMA3C induced migration of breast cancer cell lines MCF7 and MDA MB 231 in vitro (Esselens et al., 2010; Zhu et al., 2017) . However, in another study, the long form of SEMA3C did not effect migration or proliferation of MDA MB 231 cells in vitro. Furthermore, it reduced tumour formation and metastasis by MDA MB 231 cells in xenograft mouse models (Mumblat et al., 2015) . The decreased density of blood vessels in these tumours and inhibition of proliferation and VEGF signalling in endothelial cells suggest that the in vivo antimetastatic effects of SEMA3C could be related to reduced angiogenesis. We observed that SEMA3C mRNA is significantly downregulated by Notch activation in normal breast epithelial cells. Although there was no change in SEMA3C protein levels under the same conditions, this could be explained by the limited potential of total cell lysates in representing the expression of secreted proteins. Furthermore, the antibody we used to detect SEMA3C protein was not able to detect the long form specifically, which might hinder any possible effect of Notch activation.

CXCL14, a chemokine, induces proliferation, migration, and invasion of breast cancer cell lines and was found to be increased in ductal carcinoma in situ compared to normal breast tissue, indicating a protumorigenic and prometastatic role in breast cancer (Allinen et al., 2004; Pelicano et al., 2009; Rohilla et al., 2015) . CCL20, another chemokine, was also shown to trigger EMT and induce migration and invasion in MDA MB 231 and primary mammary epithelial cells (Kim et al., 2009; Marsigliante et al., 2013, 2016; Muscella et al., 2017) . Both of the chemokines were upregulated at the mRNA level in response to Notch activation in MFC10A cells and downregulated upon Notch inhibition in MDA MB 231 cells. These results suggest that Notch signalling could induce expression of CXCL14 and CCL20 to mediate its prometastatic effects. Although we failed to detect protein expression in our total cell lysates (data not shown), detailed analysis of secreted proteins could reveal whether the chemokine levels are affected by Notch signalling and therefore could trigger a paracrine or autocrine prometastatic process.

CXCR7 is a receptor of CXCL12, which is involved in breast cancer metastasis via activation of CXCR4. Overexpression of CXCR7 induces tumorigenesis and metastasis of breast cancer cell lines in vivo (Miao et al., 2007) , while its inhibition reduces the expressions of MMP2 and MMP9, which are involved in the invasion of cancer cells (Gao et al., 2015) . However, it has also been shown that CXCR7 inhibits metastasis by interfering with CXCR4– CXCL12 interaction and silencing of CXCR7 in endothelial cells results in recurrence and increased metastasis, pointing to a tumour-suppressor role of CXCR7 (Hernandez et al., 2011; Stacer et al., 2016) . In retinoblastoma, silencing of Notch ligand Jagged-2 resulted in increased CXCR7 expression (Asnaghi et al., 2016) . Our results showed that inhibition of Notch signalling via RBPjκ silencing did not effect CXCR7 mRNA level, but despite huge variation there was a tendency towards an increase in CXCR7 protein, which is parallel to what has been reported previously in retinoblastoma. However, we also observed a significant increase in CXCR7 mRNA expression in response to Notch activation, which also suggests a potential role for CXCR7 in the downstream of Notch activation in normal breast epithelial cells.

HMGA2 is a nonhistone chromatin-associated protein involved in transcriptional regulation by interfering with transcription factor–DNA interaction. In breast cancer, the presence of HMGA2 mRNA in blood and high expression in tumours are associated with poor prognosis, late stage, and increased metastasis risk (Langelotz et al., 2003; Wu et al., 2016) . HMGA2 induces migration and invasion of breast cancer cell lines via directly regulating Snail-1 expression (Thault et al., 2008; Wu et al., 2016) . We observed that HMGA2 mRNA expression is reduced by both activation and inhibition of Notch signalling in MCF10A and MDA MB 231 cells, respectively. Our results indicate that direct regulation of HMGA2 to mediate prometastatic functions of Notch signalling is unlikely. Rather, HMGA2 expression might be altered in order to compensate for the effects of Notch signalling modulation on transcription for the sake of cell homeostasis.

In conclusion, we showed that Notch signalling regulates expression of SEMA3C, CXCL14, CCL20, and CXCR7 to different extents in normal and tumorigenic breast cell lines. Investigating the functional importance of this regulation would allow us to understand whether these genes are playing a role to exert oncogenic or prometastatic functions of Notch signalling in breast cancer.

## Acknowledgement

This work was supported by a grant from the Scientific and Technological Research Council of Turkey (TÜBİTAK, 113Z088).
